# Relative Vigorous-Intensity Physical Activity Predicts Brain Microstructural Changes in Older Adults

**DOI:** 10.1093/geroni/igab046.1720

**Published:** 2021-12-17

**Authors:** Qu Tian, Jennifer Schrack, Bennett Landman, Amal Wanigatunga, Susan Resnick, Luigi Ferrucci

**Affiliations:** 1 National Institute on Aging, Baltimore, Maryland, United States; 2 Johns Hopkins Bloomberg School of Public Health, Baltimore, Maryland, United States; 3 Vanderbilt University, Nashville, Tennessee, United States

## Abstract

Physical activity especially at moderate-to-vigorous intensity may preserve brain structure in old age. However, current findings are cross-sectional and rely on absolute intensity. This study aimed to examine whether relative or absolute vigorous-intensity physical activity (VPA) predicts brain microstructural changes. We analyzed 260 initially cognitively normal and well-functioning participants(age=70.5yrs) who had VPA data via ActiHeart and longitudinal brain microstructure by DTI(follow-up=3.7yrs). Associations of VPA with microstructural changes were examined using linear mixed-effects models, adjusted for demographics. Each SD higher relative VPA defined by heart rate reserve (i.e. 21 min/day) was significantly associated with less decline in memory-related microstructural integrity, including mean diffusivity of entorhinal cortex and parahippocampal gyrus and fractional anisotropy of uncinate fasciculus and cingulum-hippocampal part, and not executive/motor-related microstructure. Absolute VPA was not associated with microstructural markers. Among well-functioning older adults, participating in VPA defined by heart rate reserve may predict less brain microstructural decline in memory-related areas.

